# Ten Years of Severe Vitreomacular Traction Syndrome without Functional Damage Demonstrated by Optical Coherence Tomography

**DOI:** 10.1155/2011/931038

**Published:** 2011-09-08

**Authors:** Michele Reibaldi, Teresio Avitabile, Maurizio Giacinto Uva, Francesco Occhipinti, Mario Toro, Marco Zagari, Andrea Russo

**Affiliations:** Department of Ophthalmology, University of Catania, 95100 Catania, Italy

## Abstract

*Introduction.* To describe anatomical and functional features in one patient with 10 years of severe vitreomacular traction syndrome (VTS) without functional damage demonstrated by optical coherence tomography (OCT). *Patient and Methods.* One patient with a history of 10 years VTS, with best-corrected visual acuity of 20/32, was followed up with OCT. Follow-up examinations, 3 months for the first year after diagnosis and every 6 months for the subsequent years, were performed. *Results.* Follow-up examinations showed no change anatomically and functionally. Far and near visual acuity was unchanged. OCT by Heidelberg Spectralis did not evidence differences from Stratus OCT images. *Conclusion.* VTS can be stable anatomically and functionally for 10 years. OCT is a valuable diagnostic tool in understanding the configuration of vitreomacular adhesion, followup, and eventually planning the surgical approach for operating on VTS.

## 1. Introduction

Vitreomacular traction syndrome (VTS) is an idiopathic disorder associated with incomplete posterior vitreous detachment (PVD). VTS has persistent vitreous traction and cystoid changes at the macula causing clinical symptoms such as metamorphopsia, micropsia, photopsia, and decreased visual acuity [1]. 

Traditionally, slit lamp biomicroscopy has been used to detect the macular vitreoretinal adhesions. However, this technique may underestimate or miss the subtle changes of vitreoretinal interface abnormalities [1].

Optical coherence tomography (OCT) has been reported to be more sensitive than biomicroscopy in identifying vitreoretinal adhesions associated with macular disease [2]. The recent introduction of ultrahigh-resolution imaging further enhances our capacity to image these entities and improves our understanding of these processes [3].

In VTS, the posterior hyaloid usually appears hyperreflective and thickened on OCT.

Although spontaneous resolution of the traction has been observed in about 11% of patients [1], surgical intervention to relieve the traction is necessary in many cases [4]. Pars plana vitrectomy has been shown to relieve the vitreomacular traction and results in visual improvement in most patients with vitreomacular traction syndrome [5, 6]. 

We report a case of severe VTS, demonstrating anatomical and functional stability for ten years shown by OCT.

## 2. Case Presentation

**Figure 1:**
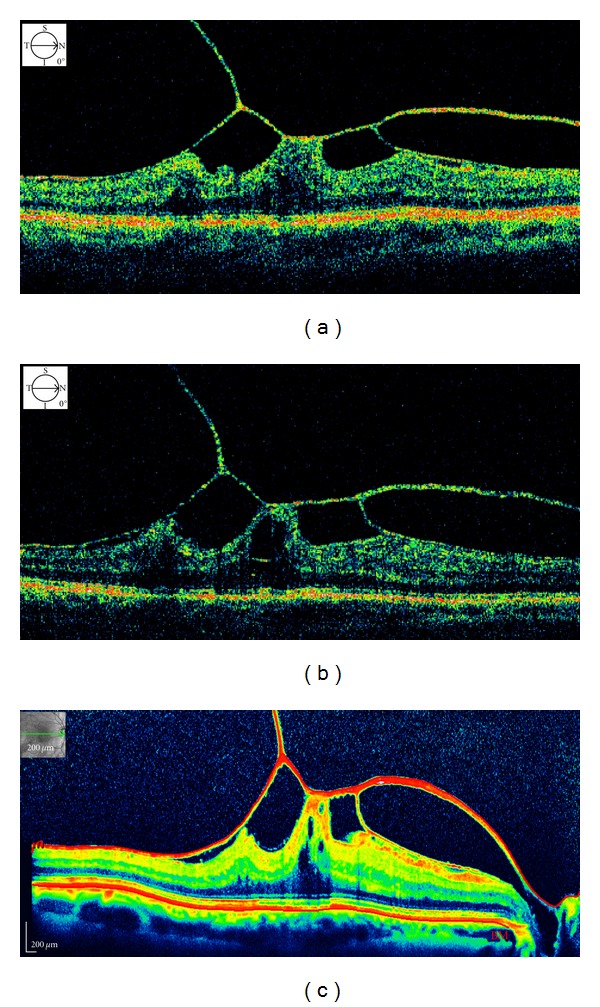
(a) At diagnosis, Stratus OCT showed a thickened hyaloid with several points of traction and cystic spaces in the internal retinal layer. Top left corner shows the direction of the scan. (b) Five years after diagnosis, Stratus OCT demonstrated unchanged features. Top left corner shows the direction of the scan. (c) Ten years after diagnosis, Spectralis OCT shows unchanged morphologic features with integrity of outer retina (photoreceptor inner and outer segment junction and external limiting membrane). Top left corner shows the direction of the scan.

A 53-year-old healthy male suffered from metamorphopsia and decreased visual acuity in his right eye for 3 weeks in 2000. He denied any ocular trauma. The anterior segment was unremarkable in both eyes. Best correct visual acuity (BCVA) was 20/32 for far and 20/32 for near in the right eye and 20/20 for far and 20/20 for near in the left eye. Biomicroscopic fundus examination revealed a dull foveal reflex with vitreous tractional maculopathy in the right eye. No Weiss ring was observed. OCT (Stratus OCT; Carl Zeiss Inc., Dublin, Calif, USA) revealed partial posterior hyaloid separation with vitreous attachment at the foveal center and parafoveally nasal and temporal, with cystic spaces in the internal retinal layer and a thin attachment to the optic nerve, resulting in macular traction (Figure 1). Central retinal thickness (CRT) was 514 *μ*m in the right eye and 191 *μ*m in the left eye. After discussing with the patient about the severity of his VTS, surgical intervention was suggested, but the patient declined and was, therefore, closely followedup. 

One month later, macular profile and thickness were demonstrated to be unchanged by OCT. The patient was then invited to effect followup every 3 months for the first year after diagnosis and every 6 months for the subsequent years. After 10 years of followup, far and near visual acuity and clinical examination were substantially unchanged, and he subjectively felt no symptoms change in his right eye. Images acquired with the new technology of OCT by Heidelberg Spectralis (Heidelberg Engineering, Heidelberg, Germany) since 2009 did not show any substantial differences from Stratus OCT images; macular vitreous traction and macular cystoid changes were not substantially different compared to the images at the diagnosis. Cross-sectional spectral OCT images shows an intact distinct back-reflection line corresponding to the photoreceptor inner and outer segment (IS/OS) junction and a thin back-reflection line corresponding to the external limiting membrane (ELM) without irregularity (Figure 1).

## 3. Discussion

We report a case of severe VTS with anatomical and functional stability for 10 years demonstrated by OCT.

VTS is one of the vitreoretinal interface disorders. The disorder is caused by incomplete PVD with persistent traction on the macula. The natural course of of VTS has been described by Hikichi et al. and associates for a median followup of five years [1]; 81% of patients had cystoid macular changes, of which 67% had persistent condition during a followup period of 60 months. The visual acuities at final examination decreased 2 Snellen lines or more from baseline in 34% of cases with VTS. Only 11% of patients with VTS developed complete PVD. To our knowledge, our case shows anatomical and functional stability for the longest follow-up. Our patient did not show spontaneous resolution of vitreoretinal traction and maintained unchanged far and near visual acuity.

Yamada and Kishi [7] described two types of partial PVD patterns. The first group was incomplete vitreous detachment nasally and temporally causing a V-shaped pattern due to persistent attachment at the fovea. The second group had persistent nasal attachment with detachment temporal to the fovea. In their study, the first group fared better postoperatively, with restoration of retinal architecture or improved vision compared with the second group. The authors hypothesized that the second pattern might result in sequelae of macular hole or macular atrophy after surgery as the uneven and chronic traction force damaged the retinal tissue, leading to subsequent development of cystoid macular edema. We hypothesize that anatomical and functional stability in our case might be related to the distribution of the tractional force, deployed to several sites that avoided foveal photoreceptor damage caused by mechanical traction, evidenziated with OCT spectral. This might explain why vitreomacular traction does not result in more severe macular damages.

 In conclusion, we have reported one case of unexpected stability of severe VTS for 10 years.

OCT is a sensitive, noninvasive and useful tool to diagnose and followup the stability of VTS. OCT by Heidelberg Spectralis (Heidelberg Engineering, Heidelberg, Germany) further improves the quality of the images and our understanding of these processes.
